# Artificial intelligence in drug discovery: what is realistic, what are illusions? Part 2: a discussion of chemical and biological data

**DOI:** 10.1016/j.drudis.2020.11.037

**Published:** 2021-04

**Authors:** Andreas Bender, Isidro Cortes-Ciriano

**Affiliations:** 1 Centre for Molecular Informatics, Department of Chemistry, University of Cambridge, Lensfield Road, Cambridge, CB2 1EW, UK; 2 Imaging and Data Analytics, Clinical Pharmacology and Safety Sciences, R&D, AstraZeneca, Cambridge, UK; 3 European Molecular Biology Laboratory, European Bioinformatics Institute, Hinxton, Cambridge, CB10 1SD, UK

## Abstract

‘Artificial Intelligence’ (AI) has recently had a profound impact on areas such as image and speech recognition, and this progress has already translated into practical applications. However, in the drug discovery field, such advances remains scarce, and one of the reasons is intrinsic to the data used. In this review, we discuss aspects of, and differences in, data from different domains, namely the image, speech, chemical, and biological domains, the amounts of data available, and how relevant they are to drug discovery. Improvements in the future are needed with respect to our understanding of biological systems, and the subsequent generation of practically relevant data in sufficient quantities, to truly advance the field of AI in drug discovery, to enable the discovery of novel chemistry, with novel modes of action, which shows desirable efficacy and safety in the clinic.

## Highlights

• Drug discovery data and data from other sources are different in quantity and characteristics.
• This article underlines the difference of data from different domains.
• In order to fully benefit from algorithms we need to address challenges posed by the data.
• Data should not be detached from a hypothesis, its representation, and the method in a data-scarce area.

## Graphical abstract

**Figure:**
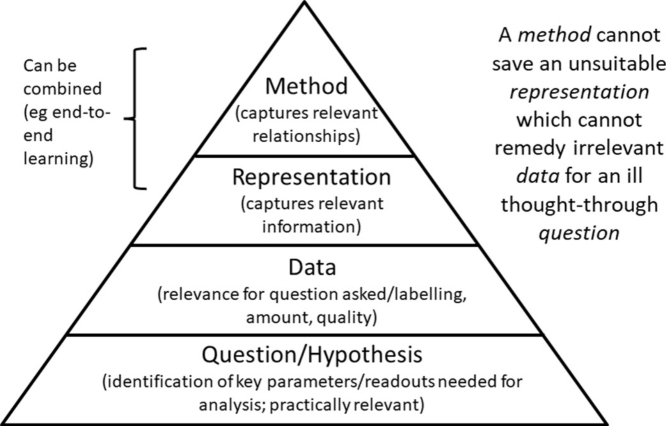

## Introduction: data in image recognition, speech classification, and drug discovery

AI has transformed many fields, probably most notably those of image and speech recognition, leading to automated passport controls and ‘virtual assistants’ (with related implications also to e.g., privacy). Focusing on the technological side from here onwards, a possible starting point of more recent developments from the image recognition side could be the 2010 paper by Schmidhuber and colleagues [1] on recognizing handwritten characters. The field rose to prominence with the 2012 NIPS paper on AlexNet [2], which successfully utilized deep neural networks for image classification. This progress was dependent not only on particular choices made by the authors [such as using successive convolution and pooling layers, using Rectified Linear (ReLU) units, data augmentation and dropout layers [3]], but also the large amounts of labeled data available from the ImageNet repository [4], as well as the use of Graphical Processing Units (GPUs). When it came to speech recognition, work such as Long Short Term Memory (LSTM) by Hochreiter and colleagues [5] paved the way for recent practical implementations and applications, such as in mobile devices and virtual assistants.

As it became clear in particular in the context of ‘deep learning’, the data available for a learning task at hand are crucial, which refers to the amount of data available, as well as their distribution (and inherent biases). The amount of data available in different domains is summarized in Table 1, comprising image data, data compiled from autonomous driving (here by Tesla), and data sets that are used in the chemical, biological, and drug discovery context 6, 7, 8, 9, 10, 11, 12, 13, 14, 15, 16, 17, 18, 19, 20. The amount of data available in different domains differs hugely, from only hundreds of data points with *in* vivo annotations (such as in case of drugs annotated with their potential to induce drug-induced liver injury, DILI) to Zettabytes (10^21^ bytes) available per year for the fleet of cars operated by Tesla. The latter is also similar to the amount of data generated by the world’s largest radiotelescope currently under development, the Square Kilometre Array, with telescopes located in Australia and South Africa. (Astronomy has long been leading the field when it comes to handling truly big data.) The amount of chemical and biological data available to use is comparatively small, in the context of other domains.

**Table 1:** Number of data points and/or sizes of data sets available in different domains, namely for images, self-driving cars, astronomy, and in the chemical, biological, and drug discovery domains^a^

Domain	Number of entries	Size of data set (bytes)	Refs
Data sets of images, from autonomous cars and astronomy			
Image Net	14 × 10^6^		[2]
Tencent image data set	20 × 10^6^		[6]
Tesla (video/sensor-derived information)		1–450 TB/day/user (assuming 2TB/day/user and 10^6^ users this means ∼2 EB/day, or ∼1 ZB/year)	7, 8
Square Kilometre Array (world’s largest radiotelescope)		∼100 TB/s (∼100 EB/year)	[9]
Data sets in drug discovery			
EMBL: raw data across databases		273 PB	[10]
REAL database of ‘drug-like molecules’	1.2 × 10^9^		[11]
ZINC database release 15 (purchasable compounds)	∼750 × 10^6^		[12]
ChEMBL: compounds with bioactivity annotations (Release 26)	16 × 10^6^	12 GB (Oracle tablespace)	[13]
Marketed Drugs (DrugBank v5.1.5)	13 548 entries (2626 approved small molecules, 1372 approved biologics, 131 nutraceuticals, >6363 experimental drugs)		[14]
DrugMatrix compound with organ-based gene expression data	627		[15]
Drugs with DILI annotations	1036		[16]
SIDER (drugs with adverse effect annotations)	1430 drugs (139 756 drug–adverse effect pairs)		[17]
Open Targets Platform (as of April 2020)	8 462 444 associations spanning 13 818 diseases and 27 700 targets		[18]
Tox21 screening data	Ca 10 000 molecules tested for 72 endpoints (∼50 × 10^6^ data points in total)		[19]
Registry of Toxic Effects of Chemical Substances (RTECS)	Data for ‘more than 160 000 chemicals’		[20]

a TB, terabytes or 10^12^ bytes; PB, petabytes or 10^15^ bytes; EB, exabytes or 10^18^ bytes; ZB, zettabytes or 10^21^ bytes.

**Figure 1:**
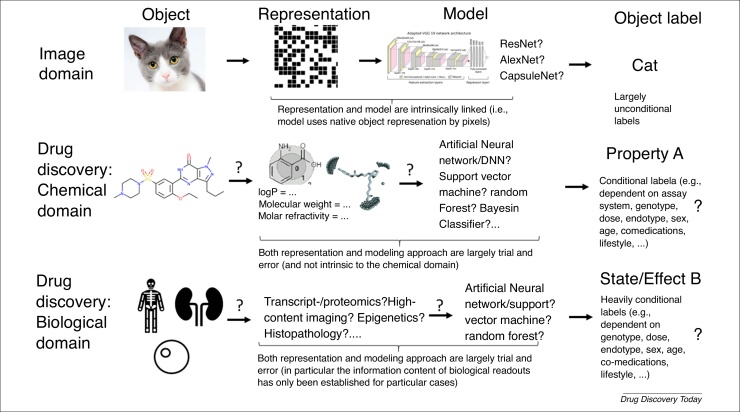
Illustration of the differences between image recognition and classification tasks in the chemical and biological drug discovery domains. When classifying images (and also speech), the model architecture and representation of object are more integrated than when using chemical and biological data, and labels can be assigned relatively less ambiguously. In the chemical domain, the best representation of an object is generally unknown (different aspects of a chemical are responsible for different types of effect, and some might be related to the functional group, others related to surface properties, etc.), whereas, in the biological domain, it is not clear which type of information provides information related to which endpoint. Common to the chemical and biological domains is that labels depend to a large extent on the set-up of a particular experiment, even if the same thing is measured ‘in principle’.

Besides the sheer amounts of data available, the ability to represent it in a computer amenable form is also vital, as is the ability to label data with relevant endpoints for data mining. A comparison between analyzing images and the domains of chemistry and biology is given in Fig. 1 for this purpose, which assumes the application of supervised algorithms in this case, where an output label for training needs to be assigned.

In the image domain, an object is usually represented by pixels (although other representations could be used [21]), which have a spatial arrangement relative to each other. Hence, objects can be visually distinguished by a computer (in the same way as by humans) insofar as they have different pixel values and, therefore, shapes or colours. This spatial arrangement of pixels is vital for image recognition using convolutional neural networks (CNNs), because convolutional layers detect object features such as edges (or more complex ones, such as faces, humans, etc.) based on particular filters that are used in the network architecture to take relative distances of the elements within images into account. In case of images, aspects of model choice also exist (with respect to both the choice of the underlying model architecture and its hyperparameters), but what is common is the agreement to use pixels as an input for such a model, which limits the possible model space. Equally, the output label of an image-based learning task can generally be assigned based entirely on the content of an image (there is no external context that needs to be taken into account) and with certainty (during human labeling; meaning either an image contains an object or not, so there is a ground truth that can be assigned unambiguously). Exceptions to this do exist, such as an image being part of a video so that labeling depends on context, objects that are hard to spot, and annotation errors in image databases. However, generally objects can be annotated based on the information visible in the image frame; there is generally little outside information that determines its labels. For image-based classification, we have a neural network available where its structure (such as convolutional layers) follows the requirements of the description of the object (its shape), and where we are able to assign labels for training generally unambiguously and on a large scale (see Table 1 for quantitative information). Analogous considerations apply to the application of recurrent neural networks (RNNs) to speech recognition (where changes of amplitude in the time domain can be appropriately captured by the structure of the network), as well as other applications where both the temporal domain and the context of the signal need to be taken into account 22, 23.

By contrast, in the chemical and biological domain, this situation of how to represent information to the computer is rather different, as shown in Fig. 1 in the middle row (see also a previous companion article for further discussions of domain differences [24]). In the chemical domain, apart from different numbers of data points present to other domains (Table 1), there are ∼3000 25, 26 known predefined descriptors, so it is by no means clear which description captures most relevant information. Some molecular properties are defined by local features (e.g., hydrogen bonds or charges), whereas others are defined by distributions of properties across the molecular surface (e.g., lipophilicity), whereas again others depend on external context (e.g., binding to a receptor, which is spatially defined, and depends on a complex balance of enthalpic and entropic factors [27]). Hence, one can choose to represent molecules as, for example, graphs, surfaces, bioactivity profiles, or using their physicochemical properties (among others). There is no lack of representations available [25]; there is only limited understanding of which representations that are predictive of the molecular property endpoint one considers in a given situation. Generally speaking, we do not know what matters about a structure for a given effect.

Given the large amount of recent work in the area of representing molecules, the representation of a molecular structure for exchanging information about its connectivity has a fundamentally different purpose from its representation for model generation purposes. The connectivity table (be it in a graph representation or, for example, in the SMILES format of a molecule) represents which bonds are present in a molecule, between which atoms. However, given that molecules are a dynamic entity (with respect to conformations, tautomerism, etc.) that exist in 3D, this representation does not capture in all cases its properties relevant for model generation. For example, a connectivity table/SMILES will not capture aspects such as the boat/chair inversion of cyclohexane (because connectivity remains identical); neither does it represent more complex stereochemistry (such as axial chirality); it can only properly represent two-electron bonds (and not, for example, three-centre-two-electron bonds); it does not capture the directionality of, for example, hydrogen bonds (and, hence, the discrimination between intramolecular interactions, and those that are accessible to interaction partners, which is crucial for aspects such as solubility); and so on. Thus, this type of information useful for exchanging the connectivity of a molecule (but which became apparent, does not cover all aspects of structural information either) needs to be clearly distinguished from a representation useful for model generation. In case of the latter, relevant properties are represented as such in the first place (such as pharmacophoric points relevant for interaction with a receptor and, hence, where external knowledge is used for their derivation, such as discussed earlier), and in this particular representation (which needs expert input in the first place!); hence, fewer data points are required for model generation. Given that data sets available for model generation are (and will be) always small compared with the size of chemical space, the requirement for suitable representations will stay with us for some time to come; that is, learned representations will probably be (and become) more useful in more data-rich areas, whereas expert-chosen representations will probably remain more useful in more data-sparse areas. In recent years, there has been some cross-over between representations that were intended to be used for exchange of structures and those used for model generation of course. In particular, use of representations used to convey connectivity of structures has been used for model generation; and this is not necessarily a bad thing either (after all, for example, MACCS keys were originally intended for cataloguing structures, but show remarkable performance in virtual screening settings, etc.) [28]. However, the above examples of limitations should be mentally distinguished, and one needs to be aware what the primary purpose of a given representation is.

In the context of recent studies, work on the ‘Molecular Transformer’ [29] has led to numerical improvements with respect to the prediction of the reaction outcome of chemical reactions by using SMILES representations of structures. Thus, given the numerical improvements, one can conclude that the use of connectivity table-derived descriptors appears to be justified empirically, because aspects such as axial chirality (one of the limitations of a SMILES/connectivity table representation) is not the primary problem we are currently confronted with. Other developments in the area of the representation of molecules have heavily focused on ‘learned representations’(e.g., 30, 31; reviewed in 32, 33). These approaches generally assign properties to the connectivity representation of a molecule, convolute those assignments, and, hence, tailor a descriptor to the particular endpoint that the model is meant to learn. In some publications 30, 31, this type of descriptor outperforms others with respect to the prediction of properties, where the dependence on sufficiently large data sets also becomes clear 32, 33, not replacing other methods with respect to every endpoint. Although no final statement shall be made here regarding the practical utility of a particular representation, the area is currently still under heavy development; it can be summarized here that endpoints can only be modeled in a meaningful way in this way where sufficient data for a relevant endpoint are available to truly learn representations and that in many cases, compared with the size of chemical space, this is not the case currently. Also, it would be necessary for performance evaluation to be performed in a way that retrospective performance translates to prospective performance, which is simply difficult with existing data sets that are often small and biased in nature (because of the heavy presence of analogs). In this regard, graph-based descriptors are naturally favored by data sets with analog bias, which makes the estimation of true prospective performance still difficult (see part 1 [24] and http://www.drugdiscovery.net/HowToLie for a more detailed discussion of model validation aspects, and the often limited utility of proxy data for the in vivo situation).

Maybe surprisingly, even (image-)matrix-based representations of molecules 34, 35, 36 perform surprisingly well, despite being far away from any chemically meaningful representation of a molecule. It is not too far-fetched to conclude that we do not understand which molecular features really matter in many cases. Hence, it is not trivial to represent a molecule to the computer for learning in the first instance, because the representation that conveys relevant information depends on the endpoint being modeled. Second, the choice of a machine-learning (ML) model is subjective and, hence, the assumption of the underlying functional form of linking molecular features to a property: for example, are features additive or do they behave in other ways? For empirically determining such relationships from the data, in most cases only insufficient amounts of data are available, given the size of chemical space and the limited data at hand (Table 1). Finally, labels in the chemical domain are very much dependent on context: is a compound toxic in the liver? This depends on dose, and the biological system a compound is tested in; or, if in the clinic, the particular individual who takes a drug. (What is the age, sex, is there any co-medication, etc.?) Is a certain nitrogen protonated, or not? This depends on the particular protein it binds to, and its dielectricity constants in the binding site (which might also change dynamically), as well as the set-up of the assay chosen (where the number of parameters is large). Even supposedly ‘simple’ molecular properties, such molecular solubility, depend heavily on the particular way one attempts to measure them [37]), as well as the particular solid it is in (amorphous, in a particular crystalline form, etc.), without even requiring information about the involvement of any biological endpoints when labeling molecules with a property. All of those aspects are not contained in the structure itself and, for many endpoints, many ways exist to measure them. In other words, the label ‘depends’, it is not defined entirely by compound structure. Hence, we are also in principle not able to label our data unambiguously. Therefore, the description of the system, devising a model, and labeling data represent problems in the chemical domain.

Similar considerations apply to modeling biological data (bottom row of Fig. 1), and probably even on a larger scale: which biological ‘descriptor’ is relevant for a problem at hand, is it one of the various -omics type of readouts? Is it, for example, transcriptomics (gene expression) where a certain phenomenon can be observed, or do we expect post-translational modification instead (or rather inactivation of the protein because of, say, a protein–protein interaction being present)? Except in very simple cases (such as overexpression of nuclear hormone receptors in some cancers), it is not trivial to understand the relationships of different levels of biology with particular endpoints of interest. Often we cannot say, because of our lack of understanding of biology and, hence, the generation of biological data is often driven by technology push, instead of science pull.

However, this difficulty does not end with the description of the system; similar aspects apply to the labeling one might want to use: How does one define, for example, ‘disease’ based on the cause, mechanism, or on the symptom level (where the levels often disagree with each other, and where some individuals with a given genetic background display very different symptoms, or also none at all) [38]? What is a drug–drug interaction; how do we define it, for example, with respect to its relationship with the dose of the drugs given, what is the impact of genotype of the individuum (some drug–drug interactions are only observed in some individuals [39]), and how do we handle frequency versus severity of events in our description? As in the case of chemical data, labeling is ambiguous and heavily situation dependent. We might be able to assign, for example, cellular cytotoxicity given a particular assay format and parameters, but whether a compound causes, for example, liver toxicity in an organism depends on the species it is tested in (and often also the strain), route of administration, dose, assay endpoint one considers, and many other factors (age, sex, co-medication, etc.). Even then, control animals also show a certain likelihood of say liver lesions in histopathology: what does one do here? Are more frequent observations of toxicity more significant, or less frequent observations, but which are observed at higher severity? In addition, as opposed to labels in the case of images, the system is not static; for example, aspects such as ‘cell line drift’ [40] exist, where the biological system evolves over time, and which has no equivalent in the image domain.

One distinction to be made here when it comes to labels in the biological domain is the difference between ‘controlled’ and ‘uncontrolled’ variability in data, with the former being, for example, the influence of a precise assay set-up, and the latter being annotations of an image chosen by a histopathologist (where, even if standardized terminology is used, annotations are often subjective). The effect of controlled variability can, in principle, be taken into account in the model, such as in multitask models (where, despite fewer data points generated in a given set-up, endpoints can learn from each other). By contrast, uncontrolled variability leads to noisy data and, hence, models with lower performance. In practice, however, this is not a black-and-white situation, such as in the case of cell line drift [40] mentioned earlier, which is supposedly a controlled part of the assay set-up (where metadata to this effect can and should be added to the assay annotation), but in real-world situations those annotations are also often not kept under control in practice.

Biological labels are hugely conditional and multidimensional and, hence, it is not trivial to assign labels in particular in case of more complex biological endpoints. Given that ML algorithms need labeled data to learn from this represents a problem when applying AI in the drug discovery field using supervised techniques, which require labels for decision-making in the first place. (Whereas unlabeled data can be subject to, for example, clustering, such as in case of single-cell data, where ample information is available [41], without insight into the meaning of individual clusters, such techniques are generally not useful for making decisions in drug discovery projects, where a decision needs to be made based on a given model output).

Thus, we can conclude that areas of previous success of AI, such as those for image classification and speech recognition, differ from the chemical and biological data available in the drug discovery field with respect to the following: (i) the amounts of data available; (ii) ability to represent it in a suitable form to the computer; (iii) a ML algorithm that is intrinsically consistent with the data available (e.g., waveforms and RNNs, or images and CNNs have this underlying consistency, which doesn’t exist for chemical and biological data); and (iv) the possibility to assign meaningful labels (which are in the drug discovery area heavily dependent on the situation, such as dose, genotype, assay set-up, etc.). Also recent other developments, such as the success of DeepMind in winning the recent CASP protein folding competition by a large margin [67] need to be evaluated in what precisely this allows us to do in the context of drug discovery. For the area of protein folding this indeed is a very important development, given the precision and speed with which predictions of folded protein states are now possible. However, for the field of drug discovery crucial questions of in vivo efficacy and safety of any drug remain as before - we will likely be able to dock (and do structure-based design) on more targets than before to discover ligands faster; how this translates into the in vivo situation is an entirely open question, and the comments above on ‘ligand’ vs ‘drug’ discovery apply as before. For a recent comprehensive discussion of the topic also see [68]. In short, there is a difference between identifying objects on images, and also predicting a protein structure, and identifying safe and efficacious drugs. We will describe the differences in more detail in the following.

We can furthermore distinguish domains depending on whether the representation of information to the computer is relevant to the objective at hand, whether it is comprehensive (i.e., whether it contains sufficient information to classify every object in principle), whether the underlying distribution of the space to be classified can be described (either analytically or by brute force), how well we can sample from the underlying distribution, whether data are conditional (or depends on factors outside the data available themselves), and to what extent it depends on quantitative effects, so that, for example, one concentration of a compound renders a compound toxic, and another (lower) one does not. This information is summarized in Table 2, including the domains of image classification and speech recognition, the games chess and GO, as well as chemical and biological data used in drug discovery.

**Table 2:** Comparison of data and representations in the image, speech, and chemistry/biology domains^a^, ^b^

Domain	Representation relevant for objective	Representation comprehensive	Underlying distribution known	Sampling of underlying distribution	Conditionality of data	Quantitative dependence of label on external context
Images	Pixels describe object (but dependent on orientation)	Yes within domain (images contain all information about visual object)	No	Biased but good (large data sets available)	Partial	None (labels can be assigned in binary fashion)
Speech	Yes (waveform captures all aspects of speech)	Yes	No	Biased and good (large data sets available)	Partial (context); local and global structure	None (words can be assigned entirely based on waveform)
Chess/GO	Yes (locations and functions of pieces are fully defined)	Yes (positions of pieces entirely describe state of system)	Can be calculated in principle, because there is a large but finite set of movements	Can be exhaustively sampled (in principle)	No	N/A
Drug discovery: chemistry	Depends on context: which features/representation of compounds is relevant is often unknown	Partially (conformations, protonation states, etc. are frequently unknown)	No (chemical space not known in its entirety; can only be calculated as approximation)	Biased and small (100 s; up to 10^6^–10^9^ out of 10^63^[49])	Partially (e.g., lipophilicity depends on protonation states, etc.)	Depends on context
Drug discovery: biology	Which aspect of biology contains information for which endpoint is frequently unknown	No (level of biological type of data generated, temporal, and spatial domain not explored)	Very partial (e.g., amino acid distributions in evolution)	Biased (depends heavily on experimental set-up)	Yes (e.g., gene expression depends on treatment, cell type, etc.	Very large (biological system is heavily influenced by system, experimental set-up)

a Given recent successes of AI in the games Chess and GO, these are included for comparison. It can be seen that chemical, and in particular biological, systems are difficult to describe, given that data can be generated on many different layers (genes, proteins, etc.); representations are not comprehensive; sampling is low; and data depend significantly on the condition of the system, while quantitative aspects abound (e.g., different concentrations of a chemical can lead to entirely different biological responses).

b The colour scheme indicates in which cases data and representations are expected to cause relatively few problems in computational models (green), an intermediate problems (yellow), or large problems (red) due to either high dimensionality, incomplete data, or incomplete definition of the problem in a given representation, or due to other reasons.

Progress in games such as GO has recently been heralded as a ‘breakthrough’ of AI [42]. Although this might well be the case in this particular domain, such games are also much simpler than a drug discovery setting, in that there is a finite set of states, rules are defined explicitly and can be calculated exhaustively (either in practice, but at least in principle). However, this is a very different set-up from the life science area: Here, in particular in the biological domain, systems generally do not follow explicitly defined rules (or at least those are generally not known, and can only be derived insufficiently from sparse data available). Rather, systems can be defined on a large number of different levels (e.g., the transcriptomics, proteomics, and metabolomics levels, but then also the epigenetic and functional interaction levels, on a time- and spatially resolved manner, taking both intracellular and intercellular signaling into account, from the cellular to the organism level). Furthermore, observations in the biological domain are highly conditional (depend on a large number of parameters) which are generally not known. In a database such as SIDe Effect Resource (SIDER) [17], one might annotate a drug with a particular side effect, and use this information to train computational models for prediction purposes. However, this effect also depends, besides the drug administered itself, on: (i) dose; (ii) genetic set-up of the recipient (such as CYP expression [43] or genetic polymorphisms [44]); (iii) factors influencing pharmacokinetic (PK) properties of the drug (e.g., food intake [45]); (iv) co-medication; (v) disease state; (vi) sex; (vii) age; or (viii) microbiome [46]; and then the side effect might only occur in a particular fraction of patients, and it might also be apparent at different levels of severity, in different forms in different organs. Ontologies used to annotate side effects might (or might not) agree with respect to term mappings and their relationships, and there might be cultural differences with respect to adverse event reporting in different populations. (Finally, it is not trivial to perform statistics based on side effect data, which suffer from profound biases [47], such as reporting bias.) As apparent from Table 2 (in addition to Table 1), data and their meaning in different areas heavily differ, and not every approach that works in one area can be directly transferred to another. We need to conclude that chemical and biological data needs to be used with significant care, and always be interpreted in its context.

The term ‘data’ is convenient in terms of thinking that ‘more data will solve our problems’ in drug discovery. However, this is not true, as described earlier, to quote Sydney Brenner, ‘Indeed, there are some who think that all that will be required is the collection of more and more data under many different experimental conditions and then the right computer program will be found to tell us what is going on in cells. […] this approach is bound to fail […]’. [48] It needs to be data than contain a signal to answer the concrete questions we ask from it, which means data supported by understanding, and solid hypotheses.

## Why representing drug discovery information for AI is difficult: linking bioactivity to adverse events

We now illustrate, based on secondary pharmacology profiling data and linking bioactivities on the protein bases to physiological outcomes, the complexities of drawing conclusions across the chemical and biological domains. Although one might as a first approximation assume that activity against a protein target is sufficient to understand (and, hence, predict) its effect in a biological system (which indeed would be convenient for AI approaches in drug discovery) this is unfortunately not the case in practice.

**Figure 2:**
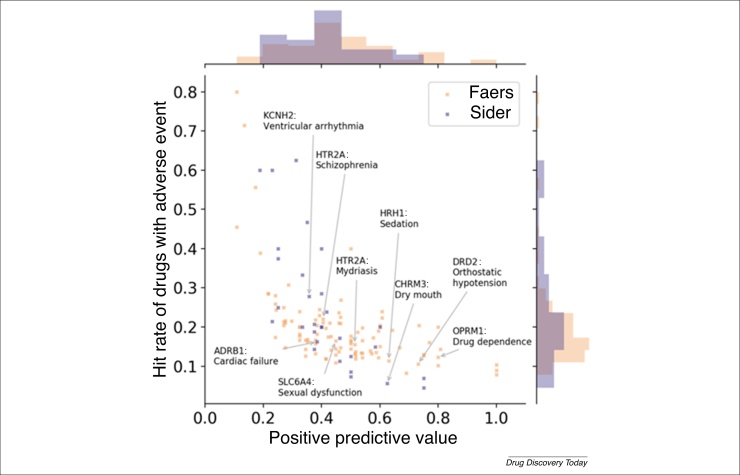
The positive predictive values (PPV) of target–adverse event associations against the hit rate or recall (i.e., the fraction of drugs associated with the adverse event also being active at an individual protein target). Activity calls were made based on the ratio of the in vitro bioactivity and the unbound plasma concentration. Target–adverse event pairs with a high PPV tend to have a low hit rate, meaning only a small share of all drugs associated with the adverse event would be picked up by the bioactivity at the target. Alternatively, a high hit rate is associated with a low PPV, indicating a high false positive rate for that target–adverse event combination. Thus, overall, there exists no clear 1:1 relationship between on-target activity and observed adverse events after compound administration. Abbreviations: ADRA1B, α1b adrenergic receptor; ACE, angiotensin-converting enzyme; CHRM1/2/3, muscarinic acetylcholine receptor M1/2/3; PTGS1, cyclooxygenase-1; DRD2, dopamine D2 receptor; FAERS, US Food and Drug Administration Adverse Event Reporting System; HTR2A, serotonin 2a (5-HT2a) receptor; HTR2C, serotonin 2c (5-HT2c) receptor; KCNH2, hERG; SIDER, SIDe Effect Resource.

We previously analyzed such relationships themselves [50] when considering the ratio of bioactivity against a target relative to unbound plasma concentration, with respect to the likelihood of a drug showing adverse events for annotations from the US Food and Drug Administration (FDA) Adverse Event Reporting System (FAERS) [51] and the database SIDER [17]. The hypothesis is that if the blood plasma concentration of a drug is above the threshold needed to act on a particular target, then one would assume to see a particular type of side effect (or, more generally, biological effect). The results of this analysis are shown in Fig. 2. Whereas bioactivity against some targets is indeed more frequently associated with particular adverse reactions than bioactivity against other targets, there is far from a 1:1 relationship between both spaces. In particular, the positive predictive value (PPV) of activity on some side effect-related targets is often low; thus, whereas activity against a protein target (at physiologically relevant concentrations) makes it more likely to observe a particular effect, activity against a target is far from a sufficient criterion for anticipating particular drug side effects.

One might argue that this analysis is rather simplistic, because many factors are not taken into account in this analysis (compound metabolism, full PK profile, such as accumulation into organs, off-target profiles, etc.), and this is indeed the case, and the point being made here: single (or even few) descriptors of a compound will not be able to anticipate the full biological complexity of drug effects, which, however, is the underlying assumption of many computational drug discovery approaches. We look at one particular compound in Box 1 to make this point clearer 52, 53, 54, 55 (this example is also repeated in [24] because of its relevance for the point we are trying to make in both contributions).

Box 1 **Why representing drug discovery information for AI is difficult: a closer look at one compound, ketamine**

Ketamine is used as both an (overall rather safe) anaesthetic, which has been approved since 1970, and a street drug. However, in 2000, its effect as an antidepressant, when dosed significantly lower than when used as an anaesthetic, was also published [52]; in addition, its bronchodilatory properties are well known. Although ketamine has long been thought to act via blocking the NMDA receptor, other NMDA blockers, such as memantine and lanicemine, have not been successful in clinical trials, hinting to a difference in their respective mode of action, which has yet to be fully understood. More recently, besides the NMDA receptor, the opioid system has also been implicated in the action of ketamine, given that naltrexone (which acts on the opioid system) influenced the effect of ketamine in one study [53] (whereas another study found the opposite [54]). Furthermore, a metabolite of ketamine was recently found to be active in animal models of depression [55], with human studies still outstanding. This case illustrates the difficulty of annotating drugs with clear mode of action and indications labels, which are often not known in detail, and depend on dose and metabolism (among other factors). This renders the application of AI approaches on such poorly labeled data in the drug discovery context nontrivial.

Alt-text: Box 1

## Data and problem settings in drug discovery

Whereas drug discovery in textbooks tends to follow a logical path, such as from target validation to hit identification, lead optimization, and into the clinical phases, this linear sequence is somewhat abstract and often shows more iterative features (or heavy tailoring to the specific situation) in practice. Nonetheless, for the purpose of using data analysis methods in the drug discovery domain, we can generally distinguish two types of model: (i) those based on large(r)-scale and proxy measures, which often aim at compound selection from a large set of either physically available or virtual molecules (e.g., in virtual screening), and which tend to be more qualitative in nature (see the first and third columns of Table 3), including models for solubility, logD, or bioactivity on protein targets usually fall into this category; and (ii) models that are based on the available data usually on a smaller scale (but which are often more relevant data for in vivo endpoints), which are of more quantitative nature, and which aim for the prediction of safety- or efficacy-related endpoints (see second and last column in Table 3). More complex models, such as animal models (or also human efficacy or safety data), fall into this category.

**Table 3:** Data available at different stages of drug discovery, and different problem settings^a^

Data availability		Problem setting	
Proxy data (usually *in vitro*)	Efficacy/safety data (usually *in vivo*)	Proxy data (usually *in vitro*)	Efficacy/safety data (usually *in vivo*)
Often ‘simple’ readouts (e.g., activity on protein) Large number of data points for training models Models have clear labels (within limits of model system; e.g., ‘ligand is active against protein at IC50 < 10uM’, logD, logS, etc. Good for model generation: many, clearly categorized data points Often less good for *in vivo* relevance	Quantitative data (dose, exposure, etc.) More complex assays to generate data, and fuzzy labels (classes ‘depend’, on exposure, e.g., multiple mutually dependent histopathological endpoints) Less, and less clearly labeled data: difficult from machine learning angle Recording complex data in format suitable for mining is not trivial; e.g., histopathology/*in vivo* endpoint data	Discovery setting: ‘find me suitable 100 s or 1000s out of 1 million’ (e.g., prioritization, screening) Qualitative predictions often sufficient Anything fulfilling (limited) set of criteria will do ‘for now’, predicting presence of something Computationally generative models often useful	Need to predict for this particular data point (molecule) Large number of criteria to rule out, based on limited data… predicting absence of ‘many things’ (e.g., different modes of toxicity) Predictive models needed (more tricky than generative; e.g., data coverage limiting) Quantitative predictions required (is this compound efficacious/toxic given the dose?)

a As it has been shown before for quantity, the quality of models for decision-making has significant impact on project success. This has profound implications for the use of predictive models in the context of AI in drug discovery, where, thus, models with sufficient inherent performance have to be used, and which are trained on endpoints *of* relevance for the in vivo situation (which is not necessarily a given for every endpoint).

Thus, drug discovery, in the way that it is frequently implemented in industry, often deals with proxy measures (see following section for details), where large numbers of data points can be generated by relatively simple types of assay. This is beneficial for the amount of data available for training (and, hence, coverage of chemical space, for example). However, in cases where proxy measures have relatively low predictivity for the relevant in vivo endpoint, the sheer amount of data is often not sufficient to generate a practically useful model. By contrast, quantitative readouts from complex biology might be more advantageous when it comes to their human in vivo relevance, although these data are more difficult to generate and model (because of the conditionality of data, an aspect discussed earlier).

There are considerable efforts taking place that aim at in vitro to in vivo extrapolation (IVIVE), in both the efficacy (e.g., related to clearance [56]) and the safety [57] contexts. However, the problem is that there is no ‘general’ mapping of proxy endpoints to a particular efficacy or toxicity that one is interested in; for example, a particular compound class extrapolates differently from one particular activity in one assay to one particular endpoint in vivo, be it for biological reasons (e.g., target expression), or PK properties (e.g., different transport, or metabolism etc.), or various other reasons. Given the large size of chemical space, in combination with the few data available across many different endpoints, it will also not be the case in the future that this type of mapping will ever be available in a comprehensive manner. That being said, localized IVIVE approaches do exist; however, they are generally tailored to the particular problem (chemical series, assay and in vivo endpoint in particular); thus, they are not generally applicable to the ‘AI in drug discovery’ field in its generality and significant manual work is required in every case. Why is the general lack of predictivity of proxy endpoints now important when aiming to use proxy measures in the drug discovery process, both generally, and also with respect to their prediction using computational methods? It has been shown before that the predictivity of assays (endpoints) used for decision making is key; to cite from a previous study [58], ‘changes in the predictive validity of screening and disease models that many people working in drug discovery would regard as small and/or unknowable (i.e., an 0.1 absolute change in correlation coefficient between model output and clinical outcomes in man) can offset large (e.g., 10 fold, even 100 fold) changes in models’ brute-force efficiency’. This is of significant importance for the utilization of predictive models for compound selection: if one uses models with insufficient quality for predicting compound properties (be it because of insufficient performance of the model itself, or an unsuitable endpoint for the in vivo situation), then a significant speed-up of individual steps in the drug discovery process will also not lead to better project outcome. Of course, the performance of models is closely linked to the quality of the underlying data, and this is often another limiting factor of using predictive models in practice [59].

To summarize, we currently do not have data of the right types available to enable us to generate models to truly utilize AI for drug discovery. Hence, incremental changes with respect to the ability to model those proxy endpoints will also not be game-changing, simply because they do not translate to any in vivo endpoints relevant to drug safety and efficacy. In many cases, we do not understand biology sufficiently well to define what we need to measure; and the choice of proxy endpoints (be they related to physicochemical properties, PK, efficacy, or safety) are always associated with significant uncertainty how they translate to the clinic. Given this property of the underlying data available, ‘AI’ approaches will also conceptually not be able to improve upon the current situation, whatever the algorithm used. (We revisit this topic in more detail in the companion article, see Table 1 in [24] and associated discussion).

## Discussion of data types and their information content in drug discovery: on data and labels in drug discovery

Although aspects of drug discovery might be intellectually understandable by humans, for modeling data using algorithms either qualitative or quantitative labels (be they binary, categorical, or numerical) need to be applied to utilize any type of explorative or predictive modeling approach. In the case of objects, these can be identifiers (‘house’, ‘car’, ‘cat’) that are often assigned manually for a training data set, and which can then be used to train a model to classify future instances without labels. Also hierarchical labeling concepts (ontologies) exist, which have been increasingly applied in the biological and drug discovery fields [60], and which have provided a helpful way to organize chemical and biological information.

However, biological annotations in particular are often far from ideal for data mining for a variety of reasons (in addition to the conditionality of biological data explained earlier), some of which are illustrated here. As an example, let us revisit the concept of a ‘mode of action’ of a drug (which has already been discussed in Box 1 for ketamine), where often Anatomic Therapeutical Classification (ATC) [61] codes are used to provide a label of this type. However, ATC codes have grown historically, and the top level category of ATC is the organ level, which is not linked to any meaningful biological mode of action. Which other options exist? For example, activity on a particular target is a common choice, say by using Entrez-Gene IDs as target identifiers. However, the situation is not as simple as that; for example, which identifier should be used if a uniquely identifiable gene is not the target, but only a particular splice variant; or a particular activation state of, for example, a kinase (e.g., the phosphorylated form); or a particular allosteric binding site? In addition, this target also might be inhibited (while its concentration remains the same), or its expression might be changed (e.g., across cell types or states), or it might be marked for degradation via PROTACs, and so on. Hence, there are many different ways of interacting with a drug target, only a few of which are functionally equivalent to each other. Furthermore, different types of interaction on the same target can lead to different effects; in the simplest case, this can be the difference between an agonist and an antagonist on a protein; but receptor pharmacology is of course more subtle than that, and biased signaling or considering the pharmacodynamics of ligand–receptor interactions, and their functional consequences, leads to further complications of how to ‘label’ a particular compound with a mode of action to enable data mining [62]. Hence, even if one accepts the premise that activity against a particular target is used to annotate modes of action of a compound (which is a big ‘if’ in itself; one can also define it say on the pathway level), then which labels to use for this purpose is by no means trivial.

An example of more complex biological readouts comes from in vivo systems, in this case histopathology information. This type of information is routinely generated in animal studies, where organs are microscopically evaluated for changes that might indicate compound toxicity. In this case, assigning observations of significance to a compound depends (besides the structure itself) on exposure (the concentration of compound in the target tissue), the subjective interpretation of observations (although this can be made more objective using image recognition techniques [63]), mutual dependency of endpoints (meaning that one endpoint has a different meaning in the context of the presence or absence of another endpoint), and the subjective use of terminology. An example of the latter are 60 different terms for ‘kidney’ encountered in the recent eTox data harmonization work of animal readouts [64], and the above list is not complete; one could also include the particular study protocol used, biological variation, and so on. Furthermore, there is the difficulty of determining which effects are treatment related and which are not (because observations also exist in the control group), and how to deal with the severity of findings [54]. In this case, histopathology endpoints are rendering this type of data nontrivial to use for AI decision-making by the very complexity (conditionality) of the data present, as well as the low amount of data that is available, which is usually only in the order of hundreds (or in the low thousands) of compounds in the largest available databases, such as DrugMatrix [15] and TG-GATEs [65]. Given the large chemical space that compounds are drawn from (and resulting low coverage), in combination with the high-dimensional readout space and the differences in experimental set-up, this type of information is very difficult to fit into AI frameworks at the current stage.

Similar considerations apply even for ‘commonly used’ terms, such as ‘disease’. This is not one biologically coherent entity one can label: it can be classified, at least, because of the underlying cause, mechanistic process, or at the symptomatic level [38]. Links between those levels depend considerably on the individual; that is, identical genetic backgrounds do not necessarily manifest themselves in the same way in different individuals. Generally speaking, if one goes from the in vitro to the in vivo area, the underlying data become more complex, smaller scale, and highly dependent on the particular set-up (protocol, individual, etc.), representing problems for AI approaches.

This inability, in many cases, to label compounds in a meaningful way leads to problems when mining data, be it in predictive models or using, for example, knowledge graphs. If we cannot represent the property and problem of interest in a suitable way, and have data from different sources (with unknown confidence), this leads to uncertainty in the predicted labels in turn. If the space we could label is large (such as 10^63^ plausible small molecules [49]), and our rate of overpredictions is nontrivial, then this leads to a large number of false positives from our model output, making it difficult to choose the output from AI systems as directly actionable input.

Table 4 provides a brief summary of such problems in the chemical, biological mode of action, and phenotypic domains for different types of representation and label currently being used.

**Table 4:** Different types of chemical and biological information utilized in drug discovery, along with a brief description of where and why labeling data is nontrivial in each domain

Data type	Representation	Difficulty/shortcoming	Resulting problem in AI applications
Chemical structure	1D descriptors, fragments, graphs, pharmacophores, surfaces, etc.	Descriptor choice subjective (not known beforehand/trial and error), etc.	No problem-inherent representation Information not necessarily contained in chemical structure and, hence, descriptor (e.g., dose-related properties, metabolites that could contribute to both desired and undesired effects, etc.)
Biological data			
Activity on protein target	Single number (e.g., IC_50_, Ki/Kd, etc.)	Functional pharmacological effects incompletely characterized Not all protein targets considered/data available for Activity on target not necessarily relevant at therapeutic dose (depends on compound PK), etc.	Ligand–protein labels are both incomplete (not available for all combinations) and heterogeneous (stem from different types of endpoint measured) Activity for isolated protein might not be relevant in *in vivo* system
Mode of action	Target, pathway, functional level, etc.	Can be defined on different levels, there is no intrinsic ‘mode of action’ of a compound Often contradictory information on different levels of readouts Depends on precise system (e.g., cell type), etc.	Labels heterogenous (because concept is not properly defined itself; see also ketamine case study in Box 1 in the main text as an example),
Gene expression data	Up- and downregulation of individual genes (or pathways)	Noisy Depends heavily on assay set-up (cell line/system, time point, dose) Wide choice of pathway annotations Wide choice of analysis methods and parameters, etc.	High-dimensional and noisy input space (∼20 000 dimensions) Relevance of gene expression changes for physiological endpoint not always clear Very parameter dependent
Cellular imaging readouts	Images themselves, or explicitly defined features	Dependent on parameters (cell line/system, time point, dose) Difficult to interpret biologically Can be difficult to identify signal (especially in hypothesis-free screening setups), etc.	Not trivial to infer biological meaning (and select relevant features) Predictive value for many practical purposes still needs to be established Very high dimensional (especially when derived in hypothesis-free set-ups)
Physiological data			
PK data	Concentration over time per tissue type	Difficult to generate (especially for tissues that are difficult to access, e.g., brain, lungs) Interpersonal variability Tracking metabolites not trivial, etc.	Generally too few PK data available (almost none for tissues) Makes it difficult to predict exposure-dependent effects (which however are crucially related to both *in vivo* efficacy and toxicity)
Animal endpoints	e.g., clinical parameters, histopathology	Biological variation Lack of consistent terminology and use of ontologies Difficult to establish treatment-related effects Frequency and severity difficult to compare Endpoints relevant only in context Time and dose dependence of observations, etc.	*In vivo* readouts difficult to label (see difficulties on the left), also generally only available on a smaller scale *G*enerally too few data to expect algorithms to infer relationships between drug treatment and *in vivo* endpoint observations
Clinical endpoints	Organ-based endpoints (eg DILI); adverse events such as in MedDRA, disease annotations such as from ICD-10, etc.	Endpoint definitions are partially overlapping and cannot be assigned clearly (e.g., DILI depends on dose etc.) Adverse events depend on reporting (with associated biases) Disease annotations depend on generally subjective evaluation by medical professionals Complete genetic background usually not known/considered (leads to incomplete knowledge about system), etc.	*In vivo* endpoints are heavily conditional, biased, and can be assigned in different ways (leading to lack of consistency among data sets) *G*enerally too few data to expect algorithms to infer relationships between drug treatment and *in vivo* endpoint observations

^a^It can be seen that little of the information in the field can easily be assigned labels, be it from the chemical or biological domain, with resulting difficulties to using such data for applying AI approaches in drug discovery.

## So where are we now, and what is the way forward for Artificial Intelligence in drug discovery when it comes to data?

The earlier discussion was a brief illustration of why it is currently not trivial to apply AI methods in the drug discovery context, which is, to a good extent, because of difficulties in generating and labeling relevant chemical, biological, and physiological data for questions related to efficacy and safety. Currently, we frequently ‘model where the data is’ (in the way of looking for the car keys where the light is, but not where we expect to find them); and real breakthroughs in the field are still scarce. Just having ‘data’ does not help (it might, to an extent, in other areas, but much less so when it comes to drug discovery): they need to be the right data, and be available in the right format and be used for the right purpose, for AI in drug discovery to bring a real change to the area.

That being said, it has been recognized that drug discovery data need to be organized much better than they have been traditionally [66], with the general trend that we are better able to do data look-up, cataloguing (where possible), and nearest neighbor searches in many cases already, all of which is useful. Still, to go to the next level, we need to go beyond the limitations posed by current data, and decide which data we need to answer a question related to in vivo safety and efficacy according to the information they contain for the question at hand.

**Figure 3:**
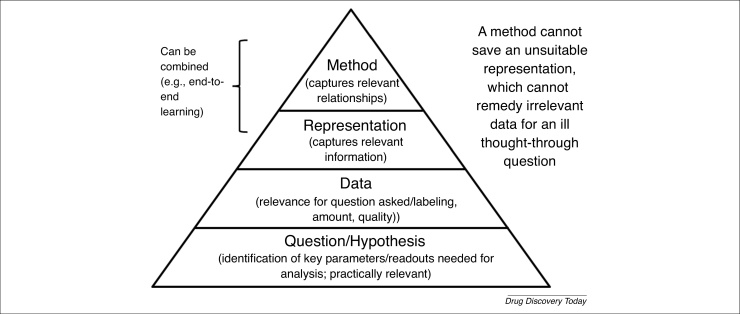
Illustration of a scientific question, or hypothesis, at the basis of data generation. The hypothesis leads to the generation of relevant data for a given question, which are represented in a signal-preserving manner; and which are then analyzed using a method that is able to handle the signal in the data. A method cannot save an unsuitable representation, which cannot remedy irrelevant data, for an ill thought-through question. This principle needs to be at the basis of data generation for making true use of ‘artificial intelligence’ in drug discovery.

Fig. 3 shows that the scientific question, or hypothesis, is at the start of any model: we have the hypothesis that, for example, the overexpression of a given gene is related to a particular disease. This allows us to generate data in a targeted manner (in this case, using gene expression detection methods), which we need to represent in a suitable way (to keep the signal in the data), and finally analyze the data with an appropriate method (which ideally is aware of the underlying distributions of data one expects in a particular area).

However, this is unfortunately not the case with data that are currently often used for drug discovery. To the contrary, data are frequently being generated in a hypothesis-free manner, followed by subsequent ‘fishing exercises’, and using constant parameters: a given cell line that is sufficiently easy to handle in a lab, and compounds are tested at a constant dose (e.g., 10 μM) and an often arbitrarily chosen time point (e.g., 12 or 24 h). This indeed makes it easier to mine data, but the relevance of data generated with such parameters for any in vivo situation is unclear.

Furthermore, data are frequently generated via techniques that are becoming available (see example of genome sequencing in the first part of this publication [24]), and which are hence expected to lead to major new insights: without asking the question whether these are the right data to generate, for a given purpose. to be able to truly use chemical and biological data for decision-making in drug discovery, we need to move beyond data that are generated by the ‘push’ of technology, toward a ‘pull’ of scientific need. Likewise, data that are generated in a hypothesis-free manner will remain difficult to analyze and to identify a signal in; thus, we need to identify better what to measure in the first place (and this might well be an iterative cycle of, for example, pilot studies to identify relevant parameters, and eventual large-scale data generation of what has been established to contain a signal from such explorative initial studies). To return to the quote of Sydney Brenner earlier, ‘Indeed, there are some who think that all that will be required is the collection of more and more data under many different experimental conditions and then the right computer program will be found to tell us what is going on in cells. […] this approach is bound to fail […]’ [48].

## Concluding remarks

The data available in the drug discovery field are fundamentally different in nature from those in other domains where AI has achieved great advances recently, such as image and speed recognition domains. Partly this relates to difficulties in defining the relevance of particular endpoints (and achieving meaningful labels for ML models to success); and partially this relates to a lack of current understanding of in particular biological systems (as well as other reasons outlined in this article). In many cases, it is difficult to label life science data (because of biological variation, dependency of labels on precise assay set-up, mutual dependence of labels on context, and inconsistent naming schemes, among others) which is a severe problem when applying AI methods in the drug discovery field. To truly advance the field, and to go from applications in ligand discovery to those in drug discovery (beyond developing chemical probes, which of course can be useful for target validation by themselves for example), we need to understand which data to generate for which purpose, and this involves understanding biology better in the first place. Only when we are then able to measure and capture relevant biological endpoints in vivo we will be able to advance the field significantly further, and to apply the computational algorithms currently available to us fruitfully in the drug discovery area, with respect to compound efficacy and safety in the clinic.

## Conflict of interest

AB is a shareholder of Healx Ltd. and Pharmenable Ltd.
